# Protocol for identification and for age assessment of unaccompanied minors

**DOI:** 10.1186/1824-7288-41-S2-A12

**Published:** 2015-09-30

**Authors:** Patrizia Carletti

**Affiliations:** 1 Observatory on Health Inequalities, Health Department, Marche Region, Italy

## Background

The national technical board, established in 2008 on request of the Marche Region Health Department, is coordinated by the Observatory on Health Inequalities and composed of members of Regional Health Departments, Health Ministry technicians and experts from the Italian Society of Medicine of Migrations; it guarantees a constant dialogue and cooperation between the Regions and the national level about issues and policies for immigrant's health care. Its aim is to encourage policy makers to fight immigrants’ health inequalities and to achieve geographical uniformity and fairness in the access to health care by the immigrants.

## Materials and methods

In 2012-2014 the board developed the “Protocol for identification and for age assessment of unaccompanied minors (UM)”. The document, issued with the contribution of Ministries of the Interior and Justice, Save The Children, UNHCR and SIP, contains the philosophy of the Protocol:

1) the implementation of a holistic and multidisciplinary age assessment of the presumed minor so replacing the medical/radiological evaluation; it is assumed that both methodologies have some degree of uncertainty but the holistic one is preferred for its complex and multidisciplinary approach;

2) it is a “unitary” document containing the operating procedures to be followed by all the actors involved in the identification procedures and age assessment of UM; its implementation will lead all the different subjects involved, such as Regional Health workers and operators of the Interior and Justice Administrations, to speak a common language and attend the same practices.

## Results

The Protocol provides for:

- description of the steps carried out by the Police for the “correct” identification of the presumed minor;

- procedures to ensure the legal protection and informed consent of the presumed minor;

- holistic and multidisciplinary age assessment - suggested only when a serious doubt remains after the identification steps and in *extrema ratio* - made in a public health service by a team composed of pediatrician, social worker, intercultural mediator, psychologist, pediatric neurologist;

- the pediatrician, together with the multidisciplinary team, will decide which tests require, using the least invasive ones.

## Conclusions

The Protocol is coherent with European Directives [1]; it follows the national and international scientific recommendations about age assessment in minors [2,3]; its application can help Italy to get out of the infringement procedures. The Protocol is currently being evaluated by the Presidency of the Council of Ministers to be converted into a specific national law. Application of the Protocol requires training for health professionals to overcome the “old practice” of using radiological examinations.
